# Pseudoaneurysm of the external carotid artery following parotitis: a case report

**DOI:** 10.1097/MS9.0000000000000130

**Published:** 2023-02-17

**Authors:** Chahed Nahal, Ghadi Nahal, Refaat Alcheikh, Mounzer Assad, Zuheir Alshehabi

**Affiliations:** a Department of Pathology; b Department of Oral and Maxillofacial Surgery, Faculty of Dentistry; c Faculty of Medicine; d Department of Pathology, Cancer Research Center, Tishreen University, Latakia, Syria

**Keywords:** case report, external carotid artery, infectious, parotitis, pseudoaneurysm

## Abstract

Pseudoaneurysms of the external carotid artery of nontraumatic causes are very rare; those of infectious causes in adults are also quite unusual and are often preceded by bacteremia. Infection-related cases such as the one described here are scarce in the literature since the complication is not often calculated or expected. We present a case report of an elderly female patient who, after dental treatment and parotitis, noticed a mass behind the right mandible. After examination, the case was diagnosed as a pseudoaneurysm of the external carotid artery of an infectious cause. Management could be by surgical intervention, but the high positioning of the pseudoaneurysm and the age of the patient were contraindications. The choice was made to avoid surgery and keep the patient under long-term follow-up; no increase in its mass was observed after 3 years of follow-up.

Highlights

- The following case describes a pseudoaneurysm of the external carotid artery that occurred after parotitis – an unusual and rare case in modern-day medicine, especially in the presence of antibiotics.
- What makes the case rare, along with its occurrence and cause, is that the age of the patient and her refusal to undergo surgery have both led to simply observing the pseudoaneurysm every 2 weeks to document its growth.
- So far, the pseudoaneurysm exhibits no abnormal growth and has remained relatively stable in terms of size.

An arterial pseudoaneurysm, also known as a false aneurysm, is caused by damage to the arterial wall, resulting in a locally contained hematoma. Unlike a true aneurysm, which involves all the layers of the vessel wall, a pseudoaneurysm shows no enlargement on any layer of the vessel wall. Instead, there is blood containment by a wall developed with the products of the clotting cascade.

Causes for pseudoaneurysms are commonly iatrogenic following endovascular procedures; noniatrogenic causes include trauma and infection, whereas pseudoaneurysms of the carotid artery usually present following trauma, it is uncommon to find a pseudoaneurysm of the external carotid artery of infectious causes.

Typically, a pseudoaneurysm of the carotid artery presents with a pulsatile mass in the neck, cerebrovascular accidents, strokes, shoulder pain caused by injury to the accessory nerve or compression of cranial nerves resulting in hoarseness of voice, dysphagia, or choking.

Infectious pseudoaneurysms are usually managed surgically since conservative treatment is ineffective.

The aim of management is preventing rupture, preventing ischemia caused by peripheral embolisms, and relief of the patient’s symptoms.

This case report has been reported in line with the Surgical CAse REport (SCARE) criteria^1^.

## Presentation of the case

The case presented here is of an 81-year-old Syrian female patient who was referred to a dental clinic for dental treatment (endodontics, exodontia, prosthodontics); she began experiencing pain and swelling behind the right mandible, accompanied by a fever, within a week of her visit.

She was referred to an otorhinolaryngologist, who ordered lab tests and an ultrasound for the right parotid gland and cervical area.

Lab test results showed normal blood count values and elevation of both C-reactive protein levels (65 mg/l) and erythrocyte sedimentation rate levels (130 mm after 1 h and 145 mm after 2 h).

The ultrasound showed signs of inflammation in the right parotid gland and enlarged lymph nodes in the cervical area, the largest on the right measuring 15 mm and on the left measuring 14 mm. Thus, the patient was diagnosed with parotitis. Medicinal treatment was given by prescribing co-amoxiclav antibiotics for 20 days.

A month later, the patient began to notice swelling both beneath and behind the right mandibular angle.

A contrast computed tomography (CT) scan for the cervical area was ordered. In the CT scan, the parotid gland was of normal measurements in size and showed no signs of inflammation; there were no signs of lymphadenopathy in both superficial and deep cervical areas. However, the contrast CT scan described a soft tissue mass with measurements of 37×44 mm. After intravenous contrast injection, the mass showed irregular radiographic density. The described mass was observed to be interspersed within the vascular structures of the external right carotid artery sheath and appeared to be near the right nasopharyngeal wall.

After the contrast CT scan, a biopsy was obtained to determine the nature of the mass, the cytology results of which showed no malignant behavior of the mass.

The patient was referred to a radiologist to have a digital subtraction angiography taken, and the report showed the appearance of an aneurysm of the right external carotid artery. The case was later referred to a vascular surgeon for diagnosis, who ordered a CT scan of the aortic arch and cervical arteries; three-dimensional reconstruction showed an aneurysm with measurements of 43×33×27 mm. No abnormal masses or lymphadenopathy were observed.

Thus, the vascular surgeon diagnosed a pseudoaneurysm of the right external carotid artery.

## Methods

This case report is fully compliant with the ARRIVE criteria^2^.

**Figure 1:**
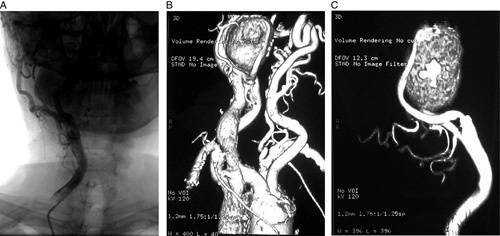
(A) Single-plane venous digital subtraction angiography of the carotid artery shows the pseudoaneurysm on the right external carotid artery; coronal view. (B and C) Three-dimensional reconstruction of the right carotid artery shows the pseudoaneurysm with measurements of 43×33×27 mm.

Surgical treatment of the pseudoaneurysm was not possible due to its high positioning in the external carotid artery. Installing a stent graft, however, was possible, but the patient’s age and refusal to undergo surgery were taken into consideration, so it was decided that the treatment would be by observing the pseudoaneurysm every 15 days by ultrasound, along with avoiding physical and emotional stress, and the mass showed stability and constant decrease in size after adhering to the treatment plan (Fig. 1).

## Discussion

Pseudoaneurysms of the carotid artery usually present following trauma, blunt, or penetrating^1^; other causes for pseudoaneurysms are commonly iatrogenic following endovascular procedures or injury during surgery^3^, radiotherapy^4^, and infection^5^,^6^. Therefore, it is uncommon to find a pseudoaneurysm of the external carotid artery of infectious causes.

The patient here had no history of surgical intervention, trauma, or radiotherapy. We can assume, therefore, that the cause of the pseudoaneurysm formation in our patient was infectious causes following parotitis. Patients may seek medical attention after noticing a mass or having complaints due to complications arising from the position and/or size of the mass.

Diagnosis can be suspected by a physician during clinical examination when presented with the typical clinical signs of the pseudoaneurysm of the carotid artery. Radiologic tests such as contrast-enhanced CT are required for determining the location, extent, and size of the mass and its relation to surrounding structures, along with angiography, which is essential for the diagnosis of such cases.

In previously reported cases, common treatments were surgical^7^ or endovascular such as a stent graft or embolization. Surgical intervention is usually recommended when convenient. Endovascular procedures are relatively safer and are associated with fewer complications.

In our case, the location of the pseudoaneurysm and the patient’s age were contraindications for both surgery and endovascular treatment, along with the absence of any clinically alarming signs or complications, so it was decided upon conservative treatment.

## Conclusion

Our case, though rarely observed clinically and rarely reported in the literature, should be put into consideration as a possible differential diagnosis when presented with typical or possible clinical signs of a pseudoaneurysm of the external carotid artery, especially in the context of recent infection in nearby tissue. It should also be noted that the measures taken to observe the case and keep its stability, instead of undergoing surgery, should also be considered as possible treatment options when presented with such cases.

## Ethical approval

This case report was conducted in accordance with the Declaration of Helsinki. The collection and evaluation of all protected patient health information were performed in a Health Insurance Portability and Accountability Act (HIPAA)-compliant manner.

## Patient consent

Written informed consent was obtained from the patient for publication of this case report and any accompanying images. A copy of the written consent is available for review by the Editor-in-Chief of this journal.

## Sources of funding

The authors received no financial support for the research, authorship, and/or publication of this article.

## Author contribution

C.N., G.N., and R.A. analyzed, interpreted the patient data, and wrote the manuscript. M.A. was the chief operating surgeon and revised the final draft. Z.A. approved the submitted version. All authors read and approved the final manuscript.

## Conflicts of interest disclosure

The authors have no conflicts of interest to declare.

## Research registration unique identifying number (UIN)

1. Name of the registry: NA.

2. Unique identifying number or registration ID: NA.

3. Hyperlink to your specific registration (must be publicly accessible and will be checked): NA.

## Guarantor

Dr Chahed Nahal.

## Provenance and peer review

Not commissioned, externally peer-reviewed.
